# Monitoring Arthropods in maize and pasture fields in São Miguel and São Jorge Islands: IPM-Popillia Project

**DOI:** 10.3897/BDJ.11.e109431

**Published:** 2023-10-05

**Authors:** Mário Brum Teixeira, António O. Soares, Paulo A. V. Borges, Mar Torres Calvet, Ángel Peñalver, Hugo R. Monteiro, Jorge Frias, Nelson Simoes

**Affiliations:** 1 Biotechnology Centre of Azores (CBA), Faculty of Sciences and Technology, University of the Azores, Ponta Delgada, Azores, Portugal Biotechnology Centre of Azores (CBA), Faculty of Sciences and Technology, University of the Azores Ponta Delgada, Azores Portugal; 2 cE3c- Centre for Ecology, Evolution and Environmental Changes, Azorean Biodiversity Group, CHANGE – Global Change and Sustainability Institute, Faculty of Sciences and Technology, University of the Azores, PT-9500-321, Ponta Delgada, Azores, Portugal cE3c- Centre for Ecology, Evolution and Environmental Changes, Azorean Biodiversity Group, CHANGE – Global Change and Sustainability Institute, Faculty of Sciences and Technology, University of the Azores, PT-9500-321 Ponta Delgada, Azores Portugal; 3 IUCN SSC Species Monitoring Specialist Group, Angra do Heroísmo, Azores, Portugal IUCN SSC Species Monitoring Specialist Group Angra do Heroísmo, Azores Portugal; 4 cE3c- Centre for Ecology, Evolution and Environmental Changes/Azorean Biodiversity Group, CHANGE – Global Change and Sustainability Institute, School of Agricultural and Environmental Sciences, University of the Azores, Rua Capitão João d´Ávila, Pico da Urze, 9700-042, Angra do Heroísmo, Azores, Portugal cE3c- Centre for Ecology, Evolution and Environmental Changes/Azorean Biodiversity Group, CHANGE – Global Change and Sustainability Institute, School of Agricultural and Environmental Sciences, University of the Azores, Rua Capitão João d´Ávila, Pico da Urze, 9700-042 Angra do Heroísmo, Azores Portugal; 5 IUCN SSC Mid-Atlantic Islands Invertebrate Specialist Group, Angra do Heroísmo, Azores, Portugal IUCN SSC Mid-Atlantic Islands Invertebrate Specialist Group Angra do Heroísmo, Azores Portugal; 6 University of Girona, Faculty of Sciences, Girona, Spain University of Girona, Faculty of Sciences Girona Spain

**Keywords:** *Popilliajaponica*, pitfall traps, biodiversity, maize, pasture, predators, *Carabidae*, *Staphylinidae*, Azores

## Abstract

**Background:**

The dataset presented here is an achievement of the H2020 European project "Integrated Pest Management of the Invasive Japanese Beetle, *Popilliajaponica* (IPM-Popillia)". This project addresses the challenge of a new risk to plant health in Europe, the invasion of the Japanese beetle, *Popilliajaponica* (Newman, 1838) (Coleoptera, Rutelidae) and provides an environmentally friendly IPM Toolbox to control the expanding pest populations across Europe. This study aims to present the records of terrestrial arthropod diversity with a special focus on four groups belonging to Carabids and Staphylinid beetles (Coleoptera), Opiliones and Anisolabididae (Dermaptera), collected with the potential to be used as biocontrol agents against *P.japonica* in future Integrated Pest Management programmes. A thorough sampling programme was conducted in maize and pasture fields in two Islands of the Azores (São Miguel and São Jorge) in the summer of 2022.

**New information:**

We provided an inventory of the arthropods recorded in two Azorean agroecosystems (maize and pasture fields) from São Miguel and São Jorge Islands. A total of ten maize and ten pasture fields were sampled and a total of 360 pitfall traps were installed, 216 in São Miguel and 144 in São Jorge, for seven consecutive days in August and September of 2022.

We collected 18559 specimens belonging to the phylum Arthropoda, four classes, twelve orders, twenty-six families and forty morphospecies (two identified at the family level as carabid and Staphylinid larvae and 38 identified at the species level). We identified 38 taxa at the species level (n = 18281). Of the 38 identified taxa, 18 species were predators, 15 were plant feeders and five were omnivores. The 18 predators belong to the following families: 10 species were Carabidae, two Staphylinidae, one Anisolabididae, one Chrysopidae, one Leiobunidae, one Nabidae, one Phalangiidae and one Scathophagidae. Concerning the origin of the predators, we recorded five native species: two Carabidae, one Leiobunidae, one Scathophagidae and one Nabidae. The other 13 predator species were introduced or indeterminate.

## Introduction

Agriculture is considered one of the most important sources of pressure for native habitats and species across Europe, driving critical biodiversity losses (European Commission, Directorate-General for Environment and Sundseth 2021). Agricultural environments in Europe are also of critical importance to the conservation of biodiversity. Indeed, these anthropogenic areas hold all wild varieties of life forms, including plant varieties and breeds of animals, soil organisms, pests and pollinators. According to the European Environment Agency, 50% of all species in the EU rely upon agricultural habitats (Kristensen 2003, European Environment Agency 2006, Stoate et al. 2009). All those species, including soil arthropods, contribute to a diverse array of ecological processes, including ecosystem services like decomposition, nutrient cycling, pollination and pest control (Willem Erisman et al. 2016, Kuťáková et al. 2018, Cuff et al. 2021, Ferrante et al. 2022), which are directly consequential for food production and security (Messer and Cohen 2007, Zarnetske et al. 2012, Ameixa et al. 2018, Ichii et al. 2019, Dainese et al. 2019, Cardoso et al. 2020, Samways et al. 2020).

While several factors contribute to the decrease in biodiversity in agroecosystems, much of this is directly related to agriculture intensification (Stoate et al. 2009). Agroecosystems with a higher diversity of soil-surface arthropods result in more efficient biocontrol (Menta and Remelli 2020, Gonçalves et al. 2020). The intensive use of pesticides can negatively disrupt arthropod populations, reducing their diversity and affecting the efficacy of biocontrol programmes. By implementing biological control conservation strategies to recruit and protect these organisms, we contribute to preserving the agricultural ecosystem, translating into safer agriculture practices (Zhang et al. 2021). Feeding more people puts additional pressure on available agricultural lands and natural areas (FoodPrint 2023, European Union 2023). The enduring challenge is the search for practices that provide new integrated strategies that explicitly balance the needs of different species and services (Tomich et al. 2011).

Regardless of their functional group, insects are a dominant component of biodiversity in most ecosystems. Ground-dwelling beetles (Coleoptera, Carabidae) and rove beetles (Coleoptera, Staphylinidae) are considered two of the most important generalist predators, having a long-standing tradition in pest management strategies in Central European agriculture. Indeed, many references assess their role as biological control agents (Luff 1987, Lövei and Sunderland 1996, Kromp 1999). In this context, any disruptive action may cause irreversible damage to the wealth and abundance of biotic communities (Samways et al. 2020) and, consequently, lead to the simplification of food webs (Derocles et al. 2018). The relatively simple architecture of agroecosystem trophic webs (Roubinet et al. 2018) may entail the loss of redundancy needed to respond to future environmental changes (Reich et al. 2012). The absence of many generalist species, that is, the lack of a redundancy system that protects against species loss, makes these systems vulnerable, for example, to biological invasions (Tylianakis et al. 2008, Heleno et al. 2020).

On the one hand, the positive contribution of biodiversity to ecosystem functioning is relatively consensual (Hong et al. 2022); on the other hand, the dependence of ecosystem services on species richness is still under debate (Dainese et al. 2019). In temperate and subtropical regions, arthropod communities present a few highly-abundant (dominant) species and many rare species (Hamilton 2005, Matthews and Whittaker 2014). This raises the pertinent question about which alternative biodiversity scenarios best favour ecosystem services, the occurrence of a small number of dominant species or many complementary species having the same functional role (Dainese et al. 2019). The few empirical studies on this issue reveal contradictory results and suggest that different agroecosystems and mechanisms underlying their functionality respond to biodiversity interactions, but are difficult to predict. A recent survey conducted by Dainese et al. (2019) discovered that, in contrast to equitability, rich and abundant pollinators and biological control agents positively impact the ecosystem. These findings suggest that dominant species play a crucial role as the primary contributors to ecosystem service.

Assessing the taxonomical and functional biodiversity of soil arthropods and their potential role in controlling pest species and the predation range of these animals are important steps in biological control. The direct observation and subsequent identification of prey fragments in the stomach of the predators is laborious and time-consuming, particularly in dynamic plant growing systems where both prey and predators have short life cycles and are of small size (Morris et al. 1999, Ellis et al. 2001, Sheppard et al. 2004, Foltan et al. 2005). However, it is possible to assess these generalist predators diets using molecular tools like Next Generation Sequencing (NGS) (Roy et al. 2021, Batuecas et al. 2022, Saqib et al. 2022).

The Japanese beetle was accidentally introduced in Terceira Island (Archipelago of the Azores, Portugal), where it became invasive. The species was first detected in the North American Air Force Base at Lajes Parish in the early 70s (Martins et al. 1988, Lopes and Mexia 1995, Lopes et al. 2001). From there, the species quickly dispersed to the other Azores islands through occasional introductions when adults or larvae were shipped through local trade. Since then, population densities have gradually increased, becoming a major pest of maize and pasture fields.

*Popilliajaponica* larvae feed on the roots of pasture grasses, which cover most of the agricultural areas of the Azores. Adults feed on leaves and flowers of hundreds of agricultural, ruderal and ornamental plant species, including maize, one of the most important fodder crops for cattle feeding (Vieira 2008). Over the last few years, there has been a higher recorded abundance of *Popilliajaponica* in São Jorge Island compared to São Miguel. Therefore, in this study, we focus on recording the epigean arthropods present on both islands to find potential predators that could be used to control the pest.

The European Project "Integrated Pest Management of the Invasive Japanese Beetle, *Popilliajaponica*" - IPM-Popillia aims to find biological control techniques to provide an environmentally friendly IPM-Toolbox to control Japanese beetles in the infested zones, protecting agricultural habitats and try to control this pest current European expansion.

In this study, we assessed the taxonomical and functional biodiversity of soil arthropods in maize and pasture fields in São Miguel and São Jorge Islands, recording generalist predators with the potential for biological control of agricultural pests. This work will provide us with the biological material to access predators diets in future work using NGS tools.

## General description

### Purpose

To provide an arthropod inventory with a focus on Carabids and Staphylinid beetles (Coleoptera), Opiliones and Anisolabididae (Dermaptera), in the agro-ecosystems of São Miguel and São Jorge Islands (Azores), based on data collected in two agro-ecosystems, maize and pasture fields. This study aims to enhance our understanding of the taxonomic and functional diversity of terrestrial arthropods, with a focus on the carabid and staphylinid groups with the ultimate goal of identifying potential biological control agents that can effectively manage *Popilliajaponica* pest populations.

### Additional information

The European IPM-Popillia project aims to control the invasion of the Japanese beetle, *Popilliajaponica*, based exclusively on environmentally friendly control measures. It is essential to implement measures to control the propagation of the insect and to contain the increase in population density to limit economic losses.

## Project description

### Title

Monitoring arthropods in maize and pasture fields in São Miguel and São Jorge Islands: IPM-Popillia Project

### Personnel

Project leaders: Mário Brum Teixeira, António Onofre Soares, Nelson Simões

Team members: Mar Calvet, Ángel Peñalver, Hugo Monteiro, Jorge Frias, Paulo A. V. Borges

Parataxonomists: Paulo A. V. Borges

Darwin Core Database Management: Paulo A. V. Borges

### Study area description

The study was conducted in São Miguel and São Jorge, two islands of the Azores (North Atlantic). São Miguel Island is situated in the oriental group (37.780411, -25.497047) and is the largest island in the Archipelago with 746.8 km² and a maximum altitude above sea level of 1103 metres. São Jorge Island is situated in the central group (38.627778, -28.017222) and is the fourth largest island of the Archipelago with 245.8 km² and a maximum altitude above sea level of 1053 metres.

### Design description

**Figure 1.**
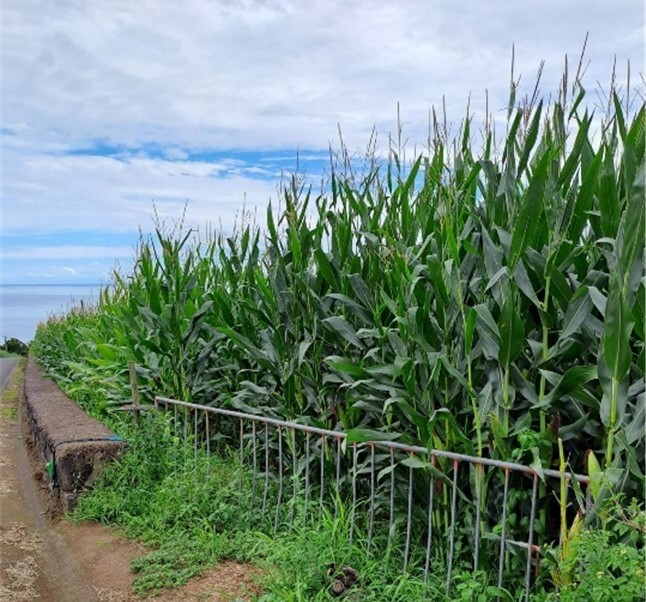
Maize field in São Jorge Island (JL1_SJ – Calheta, Ribeira seca) (Credit: Mário B. Teixeira).

**Figure 2.**
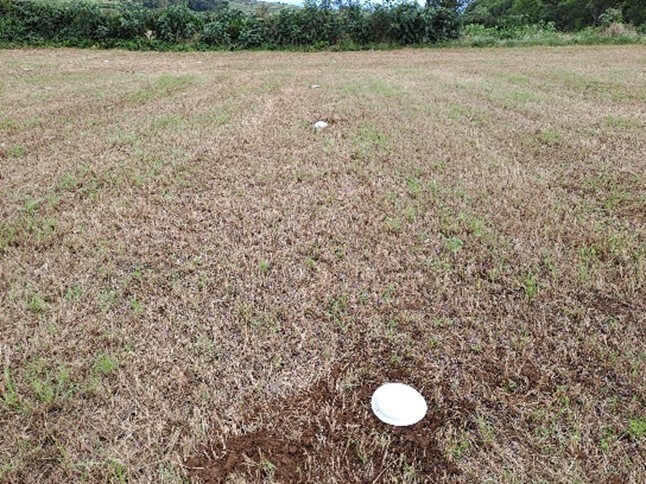
Pasture field in São Miguel Island (LRC52_SM - São Vicente Ferreira) (Credit: Mário B. Teixeira).

We sampled ground arthropods in maize fields (Fig. 1), with plants in a phenological stage of grain-filling and intensive pastures fields (Fig. 2), mainly composed of perennial ryegrass (Lolium perenne L.), annual ryegrass (Lolium multiflorm L.) and white clover (Trifolium repens L.). The sampling programme was conducted during the summer of 2022, from August to September. A total of 360 pitfall traps were installed, 216 in São Miguel and 144 in São Jorge, for seven consecutive days, when adults of Popilliajaponica were in reproductive activity, including egg laying into the soil. During this period, we also found eggs and first instar larvae in the soil and, thus, the most suitable season to record potential ground natural enemies of Popilliajaponica.

**Figure 3.**
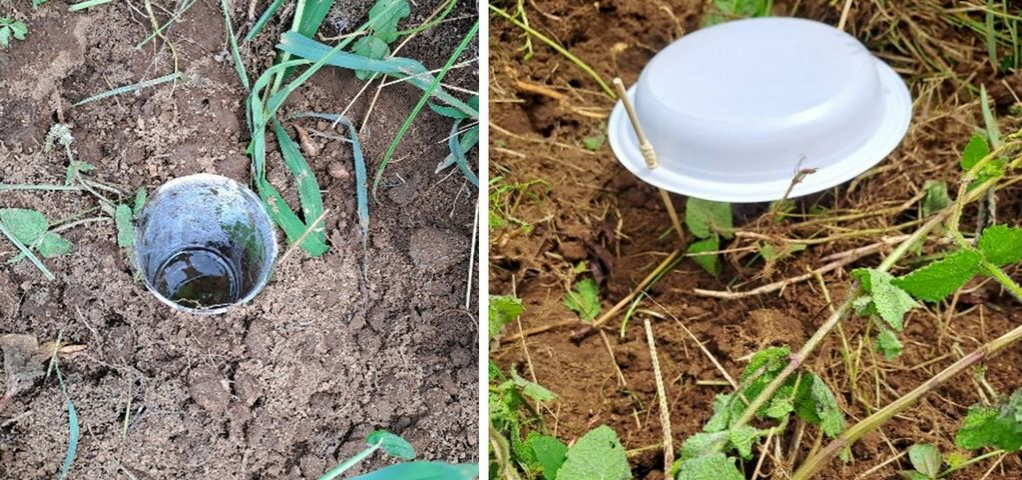
Example of a pitfall trap on the left (standard 390 ml plastic cups, 8 cm wide at the top and approximately 12 cm deep). On the right is an example of rain protection with plastic plates sitting on wooden skewers 2 cm above the ground surface (Credit: Mário B. Teixeira).

A total of 20 fields were sampled, six maize fields and six pasture fields, for São Miguel Island and four maize fields and four pasture fields for São Jorge Island (Table 1). Pitfall traps, consisting of standard 390 ml plastic cups 8 cm wide (Fig. 3), were partially filled with propylene glycol to preserve the specimens. Pitfall traps were set at the soil level to allow crawling insects to fall into the propylene glycol.

The collected specimens were sorted and posteriorly identified in the laboratory by an expert taxonomist (PAVB).

### Funding

This investigation was supported by the project IPM-Popillia: Integrated Pest Management of the Invasive Japanese Beetle, P.japonica (grant Nr. H2020-EU.3.2.1.1. / ID: 861852). M.T. and A.P. were hired by the project and J.F. received a research fellowship from the IPM-Popillia project. H.R.M. is a researcher in the CBA centre, financed by Pluriannual FCT -I.P. — Programmatic Component — Ref. UIDP/05292/2020. The student M.C. collaborated with the project under the programme of Erasmus+ Mobility for Traineeships from the University of Girona, Faculty of Sciences, Spain.

PAVB work was financed by the project Portal da Biodiversidade dos Açores (2022-2023) - PO Azores Project - M1.1.A/INFRAEST CIENT/001/2022.

## Sampling methods

### Study extent

The sampling programme was conducted in 20 agricultural fields, twelve in São Miguel (six in maize fields and six in pasture fields) and eight in São Jorge (four maize fields and four pasture fields). A total of 18 pitfall traps were installed in each site, distributed in three parallel transects. Along each transect, six pitfall traps were placed and spaced by five metres each. The transects were 25 metres long and distanced from each other by 20 metres. The transects were set from the edges to the centre of the field.

### Sampling description

Pitfall traps were used to sample ground arthropods in two agricultural habitats, maize and pasture fields of São Miguel and São Jorge Islands.

Pitfall traps consisted of standard 390 ml plastic cups, partially filled with propylene glycol and deployed for seven consecutive days.

Traps were protected from predation, inundation with rainwater and unwanted vertebrate capture (i.e. reptiles) using plastic plates on wooden skewers 2 cm above the ground surface. As the traps are sometimes fragile, two cups could be used per trap, one placed inside the other.

A total of 216 pitfall traps were installed on the 12 fields of São Miguel Island, 108 in maize fields and 108 in pasture fields. In São Jorge, a total of 144 pitfall traps were deployed, 72 in maize fields and 72 in pasture fields.

After the seven days of sampling for São Miguel, the number of pitfalls successfully recovered was 195 pitfalls, 102 in maize fields and 93 in pastures. For São Jorge, we recovered 80 pitfalls, 37 from maize fields and 43 from pasture fields.

Specimens collected were then transferred to ethanol (96%) and stored at -20ºC

Specimens were identified by Paulo A.V. Borges and Mário Teixeira, based on the Azorean arthropods collection from “Portal da Biodiversidade dos Açores, University of the Azores” led by Professor Paulo A.V. Borges. A new collection reference was created in the framework of the project IPM-Popillia, referencing each species occurring on the present dataset.

### Quality control

Before sorting, specimens were stored in alcohol (96%) at -20ºC. Specimens, adults and larvae were sorted in a laboratory by Mário Teixeira and Mar Calvet and organised in a system of morphospecies. Final identification was made by Paulo A.V. Borges.

### Step description

Final identification was made by Paulo A.V. Borges.

## Geographic coverage

### Description

The study was conducted on São Miguel and São Jorge, two islands of the Archipelago of the Azores (North Atlantic). São Miguel Island is situated in the oriental group (37.780411, -25.497047) and is the largest island of the Archipelago with 746.8 km² and a maximum altitude above sea level of 1103 metres. São Jorge Island is situated in the central group (38.627778, -28.017222) and is the fourth largest island of the Archipelago with 245.8 km² and a maximum altitude above sea level of 1053 metres.

### Coordinates

37°42'35.64''N and 38°46'47.21''N Latitude; 28°19'41.69''W and 25°7'22.75''W Longitude.

## Taxonomic coverage

### Description

The following phylum, classes and orders are covered in this study, although our scientific focus is the phylum of Arthropoda.

Phylum Arthropoda, Arachnida, Opiliones; Diplopoda, Julida, Polydesmida; Insecta, Archaeognatha, Coleoptera, Dermaptera, Diptera, Hemiptera, Neuroptera, Orthoptera; Malacostraca, Amphipoda, Isopoda.

## Traits coverage

**Taxonomic ranks**

**Phylum**: Arthropoda

**Class**: Arachnida, Diplopoda, Insecta, Malacostraca

**Order**: Amphipoda, Archaeognatha, Coleoptera, Dermaptera, Diptera, Hemiptera, Isopoda, Julida, Neuroptera, Opiliones, Orthoptera, Polydesmida

### Common names:

Bristletails, Beetles, Crustaceans, Earwigs, Flies, Bugs, Woodlouse, Millipedes, Lacewings, Opilions, Crickets, Grasshoppers, Flat-backed millipedes.

### Description:

The following phylum and orders of arthropods are covered: phylum Arthropoda and orders: Amphipoda, Archaeognatha, Coleoptera, Dermaptera, Diptera, Hemiptera, Isopoda, Julida, Neuroptera, Opiliones, Orthoptera, Polydesmida (Table 2). The individual count is organised by habitat (maize and pasture) and Island (São Miguel and São Jorge).

## Temporal coverage

### Notes

August 23, 2022 - September 30, 2022

## Collection data

### Collection name

IPM-Popillia Arthropods collection 2022. (IPMPopillia_SM_SJ_2022)

### Collection identifier

IPMPopillia_SM_SJ_2022

### Specimen preservation method

Alcohol

### Curatorial unit

Curator: Paulo A. V. Borges

## Usage licence

### Usage licence

Creative Commons Public Domain Waiver (CC-Zero)

## Data resources

### Data package title

Monitoring arthropods in maize and pasture fields of São Miguel and São Jorge Islands: IPM-Popillia Project

### Resource link

https://www.gbif.org/dataset/0a06ebda-274b-4cd7-bb13-ce449f56bb80

### Alternative identifiers

https://doi.org/10.15468/4cnhw9

### Number of data sets

1

### Data set 1.

#### Data set name

Monitoring arthropods in maize and pasture fields of São Miguel and São Jorge Islands: IPM-Popillia Project.

#### Data format

Darwin Core Archive

#### Download URL

http://ipt.gbif.pt/ipt/resource?r=ipm_popillia_azores#anchor-citation

#### Data format version

version 1.3

#### Description

The dataset table includes all the records for which a taxonomic identification of the species was possible. The dataset submitted to GBIF is structured as a sample event dataset, with two tables: event (as core) and occurrences (abundance data). The data in this sampling event resource have been published as a Darwin Core Archive (DwCA), a standardised format for sharing biodiversity data as a set of one or more data tables. The core data file contains 275 records (eventID) and the occurrences file 2226 records (occurrenceID). The data and resource metadata are available for download from Teixeira et al. (2023).

**Data set 1.** 

Column label	Column description
Table of sampling events	Table with sampling events data (beginning of table).
eventID	Identifier of the events, unique for the dataset.
stateProvince	Name of the region of the sampling site.
islandGroup	Name of the archipelago followed by the island group geographic position; oriental central and occidental.
island	Name of the island.
country	The country of the sampling site is Portugal in all cases.
countryCode	ISO code of the country of the sampling site.
locality	Name of the locality.
locationRemarks	Name of the corresponding parish.
decimalLatitude	Approximate centre points decimal latitude of the field site in GPS coordinates.
decimalLongitude	Approximate centre points decimal longitude of the field site in GPS coordinates.
minimumElevationInMetres	Approximate centre point altitude of the field site in GPS coordinates.
habitat	The habitat of the sample, only two habitats were sampled, pasture and maize fields.
geodeticDatum	The ellipsoid, geodetic datum or spatial reference system (SRS) upon which the geographic coordinates given in decimal latitude and decimal longitude are based, WGS84 in all cases.
coordinateUncertaintyInMetres	Uncertainty of the coordinates of the centre of the sampling plot.
coordinatePrecision	The precision of the coordinates.
georeferenceSources	A list (concatenated and separated) of maps, gazetteers or other resources used to geo-reference the location, described specifically enough to allow anyone in the future to use the same resources.
locationID	Identifier of the location.
samplingProtocol	The sampling protocol was used to capture the species, pitfall traps were used in all cases.
sampleSizeValue	The numeric amount of time spent in each sampling, seven days in all cases
sampleSizeUnit	The unit of the sample size value, days in all cases
eventDate	Date or date range the record was collected.
occurrence Table	Table with species abundance data (beginning of new table).
eventId	Identifier of the events, unique for the dataset.
type	Type of the record, as defined by the Public Core standard, physical object in all cases.
licence	Reference to the licence under which the record is published.
institutionID	The identity of the institution publishing the data.
institutionCode	The code of the institution publishing the data.
collection id	The identity of the collection publishing the data.
collectionCode	The code of the collection where the specimens are conserved was defined as IPMPopillia.
datasetName	Name of the dataset was defined has IPMPopillia_SM_SJ_2022.
basisOfRecord	The nature of the data record.
occurrenceID	Identifier of the record, coded as a global unique identifier.
recordedBy	A list (concatenated and separated) of names of people, groups or organisations who performed the sampling in the field.
identifiedBy	A list (concatenated and separated) of names of people, groups or organisations who performed the identification of the organisms captured.
dateIdentified	The date on which the subject was determined as representing the Taxon.
organismquantity	The quantification system used for the number of organisms.
organismquantitytype	The type of quantification system used for the number of organisms.
lifeStage	The life stage of the organisms captured were categorised in adults or larvae.
establishmentMeans	The process of establishment of the species in the location, using a controlled vocabulary: 'native', 'introduced', "indeterminate".
scientificName	The complete scientific name, including author and year.
kingdom	Kingdom name.
phylum	Phylum name.
class	Class name.
order	Order name.
family	Family name.
genus	Genus name.
specificEpithet	Specific epithet.
infraspecificEpithet	The name of the lowest or terminal infraspecific epithet of the scientificName, excluding any rank designation.
taxon rank	The lowest taxonomic rank of the record.
scientificnameauthorship	Name of the author of the lowest taxon rank included in the record.

## Additional information

We collected a total of 18559 specimens belonging to the phylum Arthropoda, in four classes, 12 orders and 26 families. A total of 40 morphospecies were identified: two at the family level and 38 at the species level. For the 38 identified taxa, we identified 18281 specimens. Although we were not able to identify the exact corresponding species of carabid (n = 34) and Staphylinid larvae (n = 244), they were also selected to further evaluate their gut contents for *Popilliajaponica*.

To meet the objectives of the IPM project, we focused on generalist predators with omnivorous behaviour. We selected four groups belonging to Opiliones (n = 4270), Carabids (n = 4370), Staphylinid beetles (n = 1697) and Anisolabididae (n = 885) specimens (Table 3). These four groups were selected, based on the species densities in the habitat sampled and their potential to predate on other live insect species as described in past research (De Heij and Willenborg 2020, Reich et al. 2020, Méndez-Rojas et al. 2021).

Considering the total specimens identified from the two Islands and within these four groups, the most abundant taxa were the native opilionid *Leiobunumblackwalli*, followed by the carabid *Pseudoophonusrufipes*, the staphylinid *Rugilusorbiculatus* and the earwig *Euborelliaannulipes* (n = 885). These most abundant species are considered voracious predators, which could act as natural controllers by reducing the densities and spread of the pest *P.japonica*.

Other carabids that showed lower densities like *Agonummarginatum*, *Agonummuellerimuelleri*, *Amaraaenea* and *Harpalusdistinguendusdistinguendus* were found to be absent or in lower densities in São Jorge Island comparing with their densities to São Miguel Island.

Amongst carabids, the dominant taxa in maize and pastures were the introduced *Pseudoophonusrufipes* and *Pterostichusvernalis*. Interestingly, these two species were more abundant in maize fields only on São Miguel Island. Within Staphylinids, the dominant taxon was *Rugilusorbiculatus* (n = 1355), more abundant in maize fields on both Islands.

In this study, we selected potential natural predators and assessed their prevalence and diversity in maize and pasture fields, which are the main habitats of the pest *Popilliajaponica* in the Azores. The invasive and native arthropods here selected will be further tested for their gut contents to unveil their feeding habits and quantify their potential for controlling *Popilliajaponica*.

We added an additional table (Suppl. material 1) of other unidentified taxa, that were not considered for the objective of this study. However, these data could be later used to access diverse food web interactions between the predators that may be associated with *Popilliajaponica* control.

### Future perspectives

This publication contributes to a better knowledge of the arthropod communities in agro-ecosystems where *Popilliajaponica* is present on São Miguel and São Jorge Islands. It will serve as a first screening to study the presence of potential soil predators for *Popilliajaponica* in pastures and maize fields of the Azores Archipelago.

## Supplementary Material

1DCFCA96-79F8-5676-B30F-FDAC76A1048E 10.3897/BDJ.11.e109431.suppl1 8416361 Supplementary material 1 Table of unidentified taxa Data type Occurrence data Brief description Occurrence data of specimens that were not in context with the objective of the manuscript and were not identified. File: oo_899756.xlsx https://binary.pensoft.net/file/899756 Paulo A. V. Borges, Mário Brum Teixeira, António Onofre Soares

## Figures and Tables

**Table 1.** Description of the habitat, locality, elevation and coordinates of the 20 sampled sites on São Miguel and São Jorge Islands, Azores.

**Habitat**	**LocationID**	**Island**	**Locality**	**DecimalLatitude**	**DecimalLongitude**	**Altitude**	**Sampling date**
pasture	FR_SM	São Miguel	São Sebastião, Ponta Delgada	37.7706010	-25.6760220	195	23-30/09/2022
pasture	AR_SM	São Miguel	Arrifes, Ponta Delgada	37.7882960	-25.7084310	263	23-30/09/2022
pasture	JS_SM	São Miguel	Arrifes, Ponta Delgada	37.7888760	-25.7085740	266	23-30/09/2022
pasture	LJB_SM	São Miguel	Arrifes, Ponta Delgada	37.7678480	-25.6842060	178	23-30/09/2022
pasture	SG_SM	São Miguel	Arrifes, Ponta Delgada	37.7807290	-25.6756220	279	23-30/09/2022
pasture	LRC52_SM	São Miguel	São Vicente Ferreira, Ponta Delgada	37.7952290	-25.6791070	250	23-30/09/2022
maize	HR_SM	São Miguel	Fajã de cima, Ponta Delgada	37.7706170	-25.6743480	199	23-30/09/2022
maize	RC53_SM	São Miguel	Fajã de Cima, Ponta Delgada	37.7939300	-25.6506190	252	23-30/09/2022
maize	JM_SM	São Miguel	Arrifes, Ponta Delgada	37.7799000	-25.6875150	259	23-30/09/2022
maize	MO_SM	São Miguel	Arrifes, Ponta Delgada	37.7808440	-25.7093820	242	23-30/09/2022
maize	PL_SM	São Miguel	Arrifes, Ponta Delgada	37.7922890	-25.6938310	281	23-30/09/2022
maize	RC52_SM	São Miguel	São Vicente Ferreira, Ponta Delgada	37.7957950	-25.6778230	245	23-30/09/2022
pasture	AC_SJ	São Jorge	Velas, Velas	38.7086260	-28.1889010	421	23-30/08/2022
pasture	JL2_SJ	São Jorge	Ribeira Seca, Calheta	38.5957420	-27.9790020	72	23-30/08/2022
pasture	MS_SJ	São Jorge	Santo Amaro, Velas	38.6920620	-28.1815500	433	23-30/08/2022
pasture	RC_SJ	São Jorge	Velas, Velas	38.7057180	-28.1987760	383	23-30/08/2022
maize	CF_SJ	São Jorge	Santo Amaro, Velas	38.6782920	-28.1640850	480	23-30/08/2022
maize	JJ_SJ	São Jorge	Santo Amaro, Velas	38.6862790	-28.1849220	348	23-30/08/2022
maize	JL1_SJ	São Jorge	Ribeira Seca, Calheta	38.5989220	-27.9720640	136	23-30/08/2022
maize	NA_SJ	São Jorge	Velas, Velas	38.6974890	-28.1955040	346	23-30/08/2022

**Table 2.** Inventory of organisms by order, sampled in maize and pasture fields in São Miguel and São Jorge Islands (Azores, Portugal) for the year 2022. The common names (Common name) and abundance values are provided.

**Rank**	**Scientific Name**	**Common Name**	**São Jorge (SJ)**		**SJ Total**	**São Miguel (SM)**		**SM Total**	**GrandTotal**
			**maize**	**pasture**		**maize**	**pasture**		
order	Amphipoda	Crustaceans	37	482	519	385	1777	2162	2681
order	Archaeognatha	Bristletails					1	1	1
order	Coleoptera	Beetles	925	1716	2641	2941	1072	4013	6654
order	Dermaptera	Earwigs	101	109	210	146	529	675	885
order	Diptera	Flyes	90	234	324	46	392	438	762
order	Hemiptera	Bugs	5	19	24	56	120	176	200
order	Isopoda	Woodlouse	80	81	161	14	55	69	230
order	Julida	Millipedes	86	75	161	25	45	70	231
order	Neuroptera	Lacewings				22		22	22
order	Opiliones	Opilions	166	216	382	1583	2305	3888	4270
order	Orthoptera	Crickets, Grasshoppers	234	481	715	637	1203	1840	2555
order	Polydesmida	Flat-backed millipedes	6	9	15	27	26	53	68

**Table 3.** List of chosen potential Popilliajaponica predators and their frequency by island and by agricultural crop, maize or pasture. Capital letters represent the means of establishment of the arthropod as I- introduced, N- native and I/N -indeterminate.

Class	Order	Family	ScientificName	Origin	São Jorge		São Miguel		Grand Total
					maize	pasture	maize	pasture	
Arachnida	Opiliones	Leiobunidae	*Leiobunumblackwalli* Meade, 1861	N	166	216	1582	2301	4265
Arachnida	Opiliones	Phalangiidae	*Phalangiumopilio* Linnaeus, 1758	I			1	4	5
Insecta	Coleoptera	Carabidae	*Agonummarginatum* (Linnaeus, 1758)	I	64		141	12	217
Insecta	Coleoptera	Carabidae	*Agonummuellerimuelleri* (Herbst, 1784)	I			127	33	160
Insecta	Coleoptera	Carabidae	*Amaraaenea* (De Geer, 1774)	I			2	14	16
Insecta	Coleoptera	Carabidae	*Anisodactylusbinotatus* (Fabricius, 1787)	I		46	28	12	86
Insecta	Coleoptera	Carabidae	*Calosomaolivieri* Dejean, 1831	N	15	196	31	43	285
Insecta	Coleoptera	Carabidae	*Harpalusdistinguendusdistinguendus* (Duftschmid, 1812)	I	1			18	19
Insecta	Coleoptera	Carabidae	*Ophonusardosiacus* (Lutshnik, 1922)	I	3	1	3	25	32
Insecta	Coleoptera	Carabidae	*Pseudoophonusrufipes* (De Geer, 1774)	I	515	707	994	271	2487
Insecta	Coleoptera	Carabidae	*Pterostichusvernalis* (Panzer, 1796)	I	107	550	307	69	1033
Insecta	Coleoptera	Carabidae	*Stenolophusteutonus* (Schrank, 1781)	N				1	1
Insecta	Coleoptera	Carabidae	*Carabidae* (larvae)	I		6	3	25	34
Insecta	Coleoptera	Staphylinidae	*Ocypusolens* (Müller, 1764)	I/N	17	11	30	40	98
Insecta	Coleoptera	Staphylinidae	*Rugilusorbiculatus* (Paykull, 1789	I/N	162	76	961	156	1355
Insecta	Coleoptera	Staphylinidae	*Staphylinidae* (larvae)	I	1	13	174	56	244
Insecta	Dermaptera	Anisolabididae	*Euborelliaannulipes* (Lucas, 1847)	I	101	109	146	529	885
